# Prey Selection of Scandinavian Wolves: Single Large or Several Small?

**DOI:** 10.1371/journal.pone.0168062

**Published:** 2016-12-28

**Authors:** Håkan Sand, Ann Eklund, Barbara Zimmermann, Camilla Wikenros, Petter Wabakken

**Affiliations:** 1 Grimsö Wildlife Research Station, Department of Ecology, Swedish University of Agricultural Sciences, Riddarhyttan, Sweden; 2 Faculty of Applied Ecology and Agricultural Sciences, Hedmark University of Applied Sciences, Evenstad, Koppang, Norway; Universita degli Studi di Sassari, ITALY

## Abstract

Research on large predator-prey interactions are often limited to the predators’ primary prey, with the potential for prey switching in systems with multiple ungulate species rarely investigated. We evaluated wolf (*Canis lupus*) prey selection at two different spatial scales, i.e., inter- and intra-territorial, using data from 409 ungulate wolf-kills in an expanding wolf population in Scandinavia. This expansion includes a change from a one-prey into a two-prey system with variable densities of one large-sized ungulate; moose (*Alces alces*) and one small-sized ungulate; roe deer (*Capreolus capreolus*). Among wolf territories, the proportion of roe deer in wolf kills was related to both pack size and roe deer density, but not to moose density. Pairs of wolves killed a higher proportion of roe deer than did packs, and wolves switched to kill more roe deer as their density increased above a 1:1 ratio in relation to the availability of the two species. At the intra-territorial level, wolves again responded to changes in roe deer density in their prey selection whereas we found no effect of snow depth, time during winter, or other predator-related factors on the wolves’ choice to kill moose or roe deer. Moose population density was only weakly related to intra-territorial prey selection. Our results show that the functional response of wolves on moose, the species hitherto considered as the main prey, was strongly dependent on the density of a smaller, alternative, ungulate prey. The impact of wolf predation on the prey species community is therefore likely to change with the composition of the multi-prey species community along with the geographical expansion of the wolf population.

## Introduction

The concept of prey selection has been central for describing the effects of predation on prey population dynamics and density [1, 2]. In single prey systems, predator kill rate will be largely dependent on prey density and therefore mainly rely on encounter rates [3]. For generalist predators living in multiple prey systems, changes in prey species density may have strong implications for their pattern of selection. True switching in the selection by the predator between prey species *sensu* Murdoch [1] will reduce predation rate on a particular species at low density and therefore can have a stabilizing effect on the system [2, 4]. However, generalist predators may also show a strict density-dependent change in their diet composition rather than true switching [5]. The pattern of prey selection by a predator may therefore have very different ecological consequences for the predator-prey community [1, 4].

Whereas all prey items available to the predators provide food and energy [6], the profitability (net energy gain/handling time [7]) provided by a specific prey depends largely on the size of the animal, i.e., the quantity and quality of biomass that it provides. A large prey could thus be more profitable than a small prey and should generally be the preferred prey within the predator’s prey size range [8, 9]. However, prey selection will also be affected by the energy that the predator spends to locate, chase, subdue and kill the prey. Similarly, the costs related to prey acquisition will also depend on the size and behaviour of both predators and prey. Although larger prey animals are more profitable, they may also cause a higher effort and risk of injury or death to the predator [10, 11, 12]. In addition, environmental conditions may also impact on the effort involved for the predator to encounter prey animals, and the efficiency by which the prey animal may escape an attack, i.e., their vulnerability [12, 13, 14].

Wolves (*Canis lupus*) are highly mobile and adaptable predators, i.e., generalists that feed opportunistically on a variety of prey species [15]. Much work on wolf-ungulate dynamics has focused on investigating wolf predation and its characteristics in relation to their primary prey species (caribou (*Rangifer tarandus*); [16], moose (*Alces alces*); [17, 18]; white-tailed deer (*Odocoileus virginianus*); [19], elk (*Cervus elaphus*); [20, 21]). However, data on prey species selection and the mechanisms linked to this process within systems of multiple prey species is less investigated (but see Huggard [10], Jędrzejewski et al. [22], Becker et al. [23], Latham et al. [24]). In general, the abundance of different prey species and their differences in vulnerability seem to be key factors in the process of prey selection for wolves [5].

Wolves are able to adjust their prey selection depending on the characteristics of the individual wolf or pack [25, 26, 27]. The motivation to kill a particular prey species may also change, depending on the time since, and the amount of biomass available from their previous kill [28]. Hence, the nutritional status of individuals may have the potential to affect prey selection. In seasonally variable regions predator selection of prey may also be affected by weather-induced effects on prey vulnerability which may change as winter proceeds [12, 28, 29, 30, 31].

Since the re-establishment of the Scandinavian wolf population [32] the distribution has mainly covered areas where the relative density of moose has been high compared to that of other ungulate species. Previous studies have confirmed that moose have been the main prey of Scandinavian wolves [33, 34, 35, 36]. Less knowledge is available on how the presence of alternative prey species will affect wolf prey selection in this region. The recent growth of the wolf population now results in that wolves are expanding into habitats having multiple prey species, mainly including roe deer (*Capreolus capreolus*), and in some cases wild boar (*Sus scrofa*), red deer, and fallow deer (*Dama dama*). This change may have large consequences for the ecology of wolves [37] as well as for the impact of wolf predation on prey population dynamics [24, 38]. The expansion of the Scandinavian wolf population offers an opportunity to study the process of prey selection and the mechanisms involved within large mammal assemblages along gradients of variable prey densities.

Here we used data from a number of geographically discrete Scandinavian wolf territories to evaluate the effects of moose and roe deer population density on inter-territorial wolf prey selection during winter. In addition, we investigated prey selection at the intra-group level by recording how wolves selected prey in relation to the spatial distribution of prey within their territory. In contrast to the first approach, the latter method gives information on how individuals on a short temporal scale respond to spatial variation in prey species abundance within their environment. We applied this novel approach by treating each kill as an observation of prey selection in relation to the spatial density distribution of the two prey species at observed kill sites. This allowed us to include additional prey-, predator-, and environment-related factors into the analyses, which improved our understanding of the mechanisms impacting the process of prey selection. Specifically, we evaluated if the current nutritional status of wolves, pack size, snow cover, and the time during winter affected prey selection of wolves. To our knowledge, this study is the first to combine such a two-scale approach to investigate the process of prey selection of a large predator.

## Materials and Methods

### Study area

The study area is the geographical breeding range of wolves in south-central Scandinavia, including both Swedish and Norwegian territories (58°50′–61°40′ N, 11°00–18°40′ E). The climate is continental with average temperatures of -5°C in January and 15°C in July [39]. During the period of study (2001–2014), average wolf population size increased from 92 (range 87–97, total count) to 400 (95% CI: 316–520) individuals [32, 40]. The biome is boreal forest and includes other large and medium-sized carnivores such as brown bear (*Ursus arctos*), lynx (*Lynx lynx*), and wolverine (*Gulo gulo*). The main prey species for wolves in this area is moose, with average winter densities of 1.3/km^2^ inside wolf territories (range 0.7–3.3) [41, 42]. In the southern wolf territories, roe deer reach average winter densities of up to 4.0/km^2^, whereas they are absent or occur at low densities in the central and northern territories. At their current distribution, red deer, fallow deer, wild boar, and semi-domestic reindeer (*Rangifer tarandus*) are not widely available to Scandinavian wolf packs. Other smaller prey species in this region are beavers (*Castor fiber*), mountain hare (*Lepus timidus*), capercaillie (*Tetrao urogallus*), and black grouse (*Lyrurus tetrix*), but constitute low amounts of the ingested biomass by wolves [34].

### Ethics statement

All procedures including capture, handling and collaring of wolves [25, 41] fulfilled ethical requirements and have been approved by the Swedish Animal Welfare Agency (Permit Number: C 281/6) and the Norwegian Experimental Animal Ethics Committee (Permit Number 2014/284738-1).

### Identification of wolf killed prey

Data on wolf prey selection on moose and roe deer were collected during 14 winters from 17 different wolf territories with wolf pack sizes ranging from 2 to 9 individuals. Seven territories were studied during two or, in one case, three subsequent years, resulting in a total of 26 territory years, hereafter referred to as predation studies (Table 1). The study period in each predation study was limited to the winter season, starting no earlier than December 11^th^ and finishing no later than May 9^th^ with the majority of study periods occurring in January through March. The mean duration of the study periods was 57.4 days and ranged from 30–132 days, resulting in a total of 1491 pack-days. Winter climate generally showed temperatures < 0°C, with a maximum snow depth of 85 cm at kill sites (mean ± 2 SE across territories = 28.3 ± 9.1 cm).

**Table 1:** Data from the 26 predation studies performed in 17 different wolf territories in Scandinavia during 2001–2014 with year and length of study, wolf pack size, territory size (100% MCP), moose and roe deer densities, and number of moose and roe deer killed by wolves. Also included is the Jacob’s index for the preference of roe deer (*D*).

Territory	Year	Pack size	Study length (days)	Territory size (km^2^)	Moose densit (km^-2^)	Roe deer density (km^-2^)	Moose kills (n)	Roe deer kills (n)	Preference (Jacob's Index; *D*)
Bograngen	2003	2	62.9	966	3.33	0.10	17	1	0.33
Djurskog	2004	5	56.2	350	1.70	0.28	13	2	-0.03
Fulufjellet	2009	6	52.0	445	1.21	0.05	8	0	-1.00
	2010	9	30.0	699	1.20	0.05	9	0	-1.00
Gräsmark	2007	6	50.5	1243	1.69	0.14	19	0	-1.00
Gråfjell	2001	2	69.3	1473	1.49	0.06	16	0	-1.00
	2002	2	132.0	1430	1.48	0.10	34	1	-0.38
	2003	6	62.6	649	2.13	0.12	24	1	-0.17
Hasselfors	2003	5	63.2	499	1.22	3.41	15	36	-0.08
Jangen	2004	2	60.0	452	0.96	0.21	13	2	-0.17
Kloten	2008	2	49.6	569	1.32	0.56	13	1	-0.69
	2011	7	49.9	754	1.33	0.45	10	0	-1.00
Kukumäki	2013	2	63.0	629	1.62	0.08	4	0	-1.00
	2014	3	53.5	776	1.61	0.07	5	0	-1.00
Nyskoga	2004	4	32.5	1284	1.34	0.06	11	0	-1.00
Riala	2010	2	42.0	164	1.37	5.84	1	39	0.80
Stadra	2003	2	50.0	837	3.32	0.73	4	16	0.90
Tandsjön	2012	5	71.0	728	0.66	0.06	12	0	-1.00
	2014	8	37.5	537	0.73	0.06	5	0	-1.00
Tenskog	2010	2	57.1	704	0.84	0.05	5	2	0.74
	2011	2	48.0	1187	0.85	0.05	6	2	0.70
Tyngsjö	2002	6	84.0	1245	1.36	0.08	22	3	0.37
Ulriksberg	2006	2	56.9	830	0.95	0.31	9	6	0.34
	2007	7	53.9	844	0.97	0.32	10	0	-1.00
Uttersberg	2006	9	61.7	381	0.89	1.69	8	0	-1.00
	2007	5	41.9	248	0.86	1.43	4	0	-1.00

To identify prey killed by wolves during the study periods we used Global Positioning System (GPS) collars for wolves (GPS-Simplex or Tellus, TVP Positioning, Lindesberg, Sweden and GPS-Plus Vectronic Aerospace, Berlin, Germany). Wolves were darted from helicopters and collars were fitted according to the methods described elsewhere [25, 41]. The collars were programmed so that at least one of the adult collared wolves in each territory recorded locations at 30 or 60 minutes intervals and data were downloaded either through a VHF-link or automatically transferred using the Global System for Mobile Communication (GSM) network.

The use of GPS data and applications with GIS for searching, finding and classifying killed prey followed the procedures described elsewhere [33,43]. Although difference in the size of prey species may under some circumstances result in variable detection rates and biased results we argue that this potential problem is of minor importance for the results in the current study. This is because 1) our high frequency positioning schedule combined with intensive field search on snow likely assured that the majority of larger prey items such as ungulates were found, and 2) even if the frequency of kills of the smaller prey species was underestimated this probability should be equally distributed among wolf territories. Pack size was estimated from intensive monitoring of GPS collared wolves on snow throughout the winter [44].

### Prey population density

Ungulate densities during winter within the 17 studied wolf territories were estimated based on counts of new pellet groups (produced since leaf fall previous autumn) during spring (between snow melt and the onset of vegetation) according to methods described elsewhere [35, 37, 45]. In each territory, a grid of 1 x 1-km square plots was systematically distributed over the wolf territory using 100% MCP from wolf GPS-locations (50–100 plots per territory). Each square plot contained 40 circular sub-plots along its perimeter, each of them covering 100 m^2^ for moose and 10 m^2^ for roe deer. The smaller sub-plot area was used for roe deer pellet group counts as the search effort increased due to the small pellet group size in roe deer. Winter density of moose and roe deer (individuals / km) was estimated by dividing mean pellet group counts for all sample plots (n_mean_: 2324, n_range_: 850–4159 / territory) by period of accumulation (days between autumn leaf fall and spring field count ranged 198–231 days) and an assumed winter defecation rate (number of pellet groups produced per individual per day) of 22 / day for roe deer [46] and 14 / day for moose [47]. The number of ungulates (*N*_*m*_) within wolf territories was estimated using the formula:

$$N_{m}=N_{p}/d\times N_{d}$$ (1)

where *Np* was equal to the total number of new pellet groups found, *d* was daily defecation rate, and *N*_*d*_ was the number of days during winter for which new pellet groups could be accumulated. For the eight territories where we performed studies of wolf predation during two or three winters, we used the same estimate of prey density for all study years. A previous study, Månsson et al. [48] tested and confirmed the reliability of the method for describing relative density variation in relation to habitat selection of moose. The estimates of intra-territorial prey population densities at kill sites were derived from interpolation of data from the systematically distributed pellet count survey, using the Thiessen polygon method [36, 49]. Prey density estimates included in the model correspond to the interpolated estimate at each kill site. Kill sites outside of the pellet count survey area were excluded from the analysis.

### Snow depth

Because snow depth was not estimated for all kill sites, we used estimates on snow depth from the Swedish Meteorological and Hydrological Institute [50] and from the Norwegian Meteorological Institute web portal eKlima [51], measured at meteorological stations at ≤ 5 km distance from the wolf territory. We identified the most appropriate meteorological station to use for each territory by calculating the 100% Minimum Convex Polygon using all wolf positions during the study period. For most data, snow depth included in analyses corresponded to the snow depth recorded at meteorological stations at the date of each kill. To estimate the snow depth for kill dates where no snow depth data was available (generally measured on a daily basis but for some stations only twice per month) we calculated the mean of the last measure before and the first measure after the actual date of kill. When snow measures were no longer available during a winter after the kill date, we assumed a zero snow depth.

### Inter-territorial differences in prey preference

To evaluate the preference of a certain prey over another at the population level (inter-territorial) we applied the Jacobs Index of Food Selection [52] to the data set using each wolf territory as one observational unit. Prey availability in the territory was equal to the total number of prey as calculated from a combination of prey population density and the size of the wolf territory. The occurrence of a certain prey type in the diet was based on the total number of verified moose and roe deer killed in the same territory during the study period. The Jacobs’ Index (*D*) was calculated as:

D=(r−p)/(r+p−2rp) (2)

where *r* is the proportion of kills of one species out of the total number of prey killed and *p* is the proportional availability of the same prey species. This index provides a symmetrical scale from -1 to 0 for avoidance and from 0 to 1 for preference. Values around zero show a neutral prey selection and we considered values from -0.5 to 0.5 as being close to neutral [53].

In addition, we used logistic regression to investigate the relationship between the proportion of roe deer in wolf-killed ungulates and; 1) the proportion of roe deer in the estimated availability of ungulates within the wolf territory; 2) roe deer density, and; 3) moose density. We also used logistic regression for investigating the relationship between Jacobs′ Index and roe deer population density and pack size. Because the data on roe deer densities at the territory level was skewed right, this variable was log-transformed. In one of the territories (Tenskog), roe deer were not detected in the pellet count, even though roe deer killed by wolves were recorded there. As roe deer apparently had been present, we assigned the lowest roe deer density registered in any other territory to this territory (0.05 km^-2^). In all models, we included pack size as a categorical variable with two classes, i.e., pair or pack of minimum 3 wolves. We weighted the observations with the length (number of days) of each study period.

### Definitions of terms of prey selection

We defined prey selection as the process where a predator selects different types of prey without reference to the abundance of particular types of prey available whereas prey preference was defined as when a predator selects prey types disproportionately to its abundance in the environment [54]. Prey switching according to Murdoch [1] occurs when the number of attacks on a prey species is disproportionally large at high prey abundance relative to other prey species and disproportionally small when the species is relatively rare whereas prey vulnerability results from a combination of capture efficiency and profitability relative to risk [12].

### Prey selection at the intra-territorial level

Although the preference indices can give us an indication of the wolves’ preference for a certain prey species and show inter-territorial differences in prey preference, it does not include other potentially important factors that may affect prey selection. The fitting of a Generalised Linear Mixed Model (GLMM) allows for the inclusion of multiple predictor variables that may affect the prey selection of wolves. Because we had only two alternative prey species the response variable had a Bernoulli distribution and we therefore fitted a GLMM with a binomial distribution and logit function (lme4 package in R 3.1.2 [55]). We used a subsample of the total dataset including 365 wolf kills including moose (n = 258) and roe deer (n = 107) from the 16 (one territory excluded: Tenskog) wolf territories for which we could obtain estimates of local (kill site) prey densities and data on snow depth. Because ungulates suffer from reduced nutritional status during winter [31, 56], we included the number of days from the first of January in the model to account for any potential effects of the winter other than snow depth. In addition, the current nutritional status of the wolf pack may also affect the prey selection. The nutritional status is a function of the edible biomass of the previous prey, the number of days since the previous prey, and the daily field metabolic rate of the pack according to the formula:

$$NS=B_{pk}-T_{pk}\times FMR$$ (3)

where *NS* is nutritional status of the pack, *B*_*pk*_ is the amount of edible biomass of previous prey, *T*_*pk*_ is the time in days since last kill, and *FMR* is the daily field metabolic rate of the pack. We used estimates of edible biomass of moose and roe deer and pack-size specific daily *FMR*s [36] to estimate the nutritional status of each pack at any time.

Scandinavian wolf territories may differ in several ways, e.g., there are latitudinal and altitudinal differences in size, variation in habitat, human population density, pack size, and other pack-specific differences. Therefore, each pack may have a different starting point for their prey selection. Using a GLMM that included the territory id as a random factor allowed an analysis of prey selection whilst allowing the intercept of the model to vary between territories, yet assuming that the other factors have the same effect on the prey selection in all wolf packs [57]. We built 10 a-priori candidate models including 1) intercept only, 2) covariates only (snow depth, day number, nutritional status), 3) the main predictors of interest only (local moose and roe deer density, additive or as interaction), and 4) a combination of main predictors and covariates. We used AICc model selection and recognised all models with a ΔAIC < 4 as equally supported [58]. For predictions, we used model averaging and estimated SE with n = 17, corresponding to the number of territories in the random structure of the models.

## Results

The full dataset included a total of 409 wolf-killed ungulates consisting of 297 (73%) moose and 112 (27%) roe deer. The proportion of roe deer out of all ungulate wolf kills was on average 15% and ranged between 0% and 97% between wolf territories. The proportion of available roe deer out of both ungulate populations was on average 19% across all territories and ranged between 0% and 81%.

### Preference at the inter-territory level

**Fig 1:**
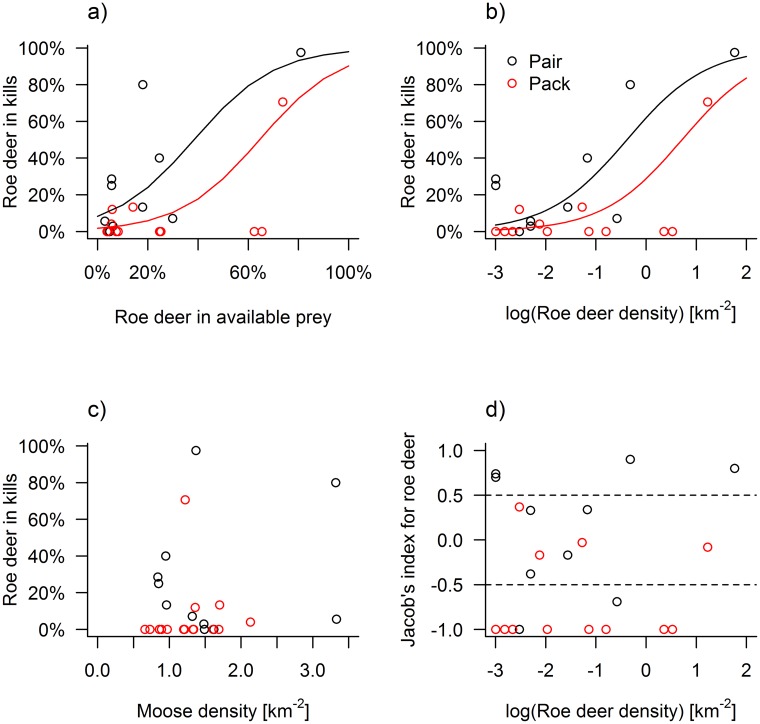
The proportion of roe deer out of the total number of roe deer (n = 112) and moose (n = 297) killed by pairs (black) and packs (≥3, red) of wolves for 26 predation studies in 17 wolf territories in Scandinavia in relation to (a) the estimated proportion of roe deer abundance, (b) the estimated density of roe deer, and (c) the estimated density of moose. Fig 1d plots the preference for roe deer in terms of the Jacob’s Index (D) in relation to roe deer density. Stippled lines indicate threshold values, with avoidance D < -0.5 and preference D > 0.5.

The Jacob’s Index showed that wolves preferred (D > 0.5) roe deer over moose in four (15%) of the 26 studies (Table 1), neither preference nor avoidance in 8 studies (31%), and avoidance of roe deer (D < -0.5) in 14 studies (54%). The proportion of roe deer out of all wolf-killed ungulates increased with the proportion of roe deer available in the environment (logit slope ± SE = 6.258 ± 1.632; χ_21,24_ = 68.63; p < 0.001) and was higher for wolf pairs than for packs (χ_21,23_ = 9.85; p = 0.002) (Fig 1A). The model predicted that pairs killed a 2.3 times higher proportion of roe deer than packs in wolf territories where roe deer made up 50% of the total ungulate abundance (Fig 1A). Models relating the proportion of roe deer in kills to the estimated densities of the two ungulate species was positively correlated with roe deer density (χ_21,24_ = 81.12; p < 0.001, Fig 1B). Model results showed that wolves on average killed more roe deer than moose at roe deer densities >1 km^-2^ and that roe deer constituted >70% of all kills at roe deer densities >3 km^-2^ (Fig 1B). In contrast, there was no correlation between the proportion of roe deer kills and moose density (χ_21,24_ = 0.32; p = 0.573, Fig 1C). Finally, prey preference in terms of the Jacob’s Index was not related to either roe deer density (χ_21,24_ = 0.25; p = 0.616, Fig 1D) or pack size (χ_21,23_ = 2.61; p = 0.106).

### Prey selection at the intra-territorial level

**Fig 2:**
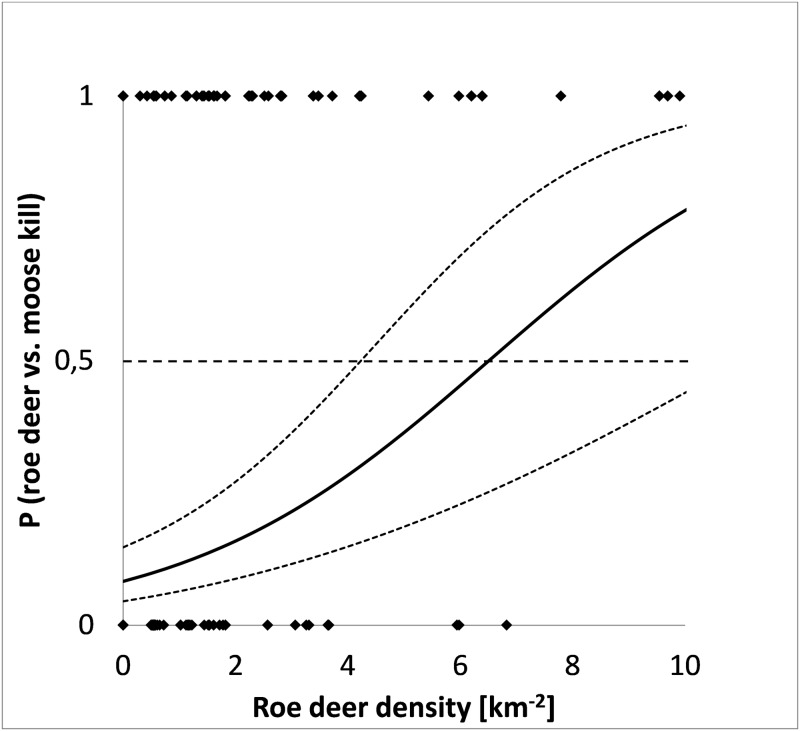
The probability of killing roe deer or moose (± SE) by wolves in Scandinavia as related to local (intra-territorial) variation in roe deer density at kill sites (n = 365) at mean moose density (1.8 / km^2^). The stippled line (Y = 0.5) indicates equal probability for wolves to kill roe deer or moose. Dots represent either moose kills (Y = 0) or roe deer kills (Y = 1).

The nutritional status of the wolf pack at the time of kill did not improve model fit of prey selection and was therefore removed from the analyses. This allowed us to increase sample size (n = 365) by also including the first kill of each study period. Local roe deer density (at kill sites, mean: 1.1 km^-2^ ± 0.3 (95% CI), range: 0–11.4) was the most important factor affecting wolf prey selection at the intra-territorial level (Tables 2 and 3). Local moose density (mean 1.8 km^-2^ ± 0.2 (95% CI), range: 0–10.6) in addition to or in interaction with roe deer density was also retained in some of the best models (Table 2). These models predicted that wolves were equally likely to kill roe deer or moose (Y = 0.5) at a roe deer density of 6.5 km^-2^ (Fig 2). At local densities higher than 6.5 roe deer km^-2^, wolves were more likely to kill roe deer, whereas at lower roe deer densities moose became the most likely kill. Mean roe deer density was 9.1 times higher at roe deer kill sites (2.86 ± 0.31 (SE) km^-2^) than at moose kill sites (0.32 ± 0.06 km^-2^), while mean moose density was only 1.5 times higher at moose kill sites (2.03 ± 0.11 km^-2^) than at roe deer kill sites (1.38 ± 0.12 km^-2^). Snow depth and time during winter were not important predictors of wolf prey selection (Table 2).

**Table 2:** Generalised linear mixed models (binomial logit) describing the Scandinavian wolves’ selection of roe deer (1) as prey over moose (0) as a function of local roe deer density (Roe deer), local moose density (Moose), winter day number (Day) and snow depth (Snow). Wolf territory is included as a random factor and the number of parameters in the model is indicated by *K*, AIC corrected for small sample size AIC_c_, differences between models ΔAIC, the model weights AIC_c_Wt, and the log likelihood value for each model LL.

Predictors	K	AICc	ΔAICc	AICcWt	LL
Roe deer	3	245.46	0	0.55	-118.73
Roe deer+Moose	4	247.36	1.9	0.21	-117.86
Roe deer*Moose	5	248.76	3.3	0.11	-116.38
Roe deer+Day+Snow	5	249.72	4.25	0.07	-116.86
Null (intercept only)	2	251.48	6.01	0.03	-123.28
Moose	3	252.31	6.84	0.02	-122.15
Roe deer+Moose+Day+Snow	6	253.65	8.19	0.01	-116.16
Day+Snow	4	254.26	8.8	0.01	-121.31
Moose+Day+Snow	5	256.75	11.28	0	-120.37
Roe deer*Moose+Day+Snow	7	257.16	11.69	0	-114.58

**Table 3:** Parameter estimates after model averaging from the most parsimonious models (ΔAICc < 4, Table 2) predicting the selection of roe deer (1) over moose (0) as kills for wolves in Scandinavia, in relation to roe deer density (Roe deer) and moose density (Moose).

Parameter	Estimate	SE	Z-value
Intercept	-2.397	0.647	-3.706
Roe deer	0.370	0.144	2.561
Intercept	-2.089	0.683	-3.059
Roe deer	0.370	0.149	2.490
Moose	-0.173	0.134	-1.290
Intercept	-2.284	0.693	-3.295
Roe deer	0.694	0.273	2.543
Moose	-0.073	0.144	-0.510
Roe deer*Moose	-0.179	0.106	-1.687

## Discussion

The current study expanded the range of available prey species as compared to previous studies [5, 33, 34, 36] by also including wolf territories that offered an important alternative prey species to moose such as roe deer. Density of this smaller prey species showed to be the most influential factor affecting prey selection of Scandinavian wolves and this pattern held at both the inter- and intra-territorial scale. In contrast, moose population density had a weak, negative relationship to selection of roe deer over moose at the intra-territorial scale. Although our findings indicate density-dependent prey switching in Scandinavian wolves, they do not support *true* prey switching *sensu* Murdoch [1969], with the relationship between the proportions of prey species killed versus available close to a 1:1 ratio.

### Prey selection at the inter- and intra- wolf territorial levels

Our two spatial levels for studying prey selection of wolves yielded similarities, but also differences. Both approaches showed that prey selection of roe deer over moose was positively associated with population density of roe deer. At the inter-territorial level, there was no clear density threshold where wolves selected roe deer, but the logistic regression model showed that roe deer tended to dominate the diet at roe deer densities >1.0 km^-2^ (Fig 1B). However, because our data were biased against areas with a relatively low density of roe deer we caution against making strong inferences about the exact relationship between prey selection and density of roe deer at the inter-territorial level. In contrast, our data at the intra-territorial level showed a more complete representation from low to high relative roe deer densities. At this level, the density threshold for selecting roe deer over moose (P > 0.5) occurred at local roe deer densities that were more than six times higher than at the inter-territorial level. This difference in threshold levels of prey density likely stem from the structure of the data and the way the analyses were carried out with estimates at the intra-territory level including only actual kill sites found during the study period. However, the results from this study show that the process of prey selection occurs at both the population scale, i.e., among packs, and at the individual or pack scale where wolves may respond to spatial variation in prey species distribution within the territory.

One potential explanation to the observed pattern is that the profitability or vulnerability of roe deer increases with roe deer population density simply because of increased encounter rates with wolves. Due to their small size (relative to moose) roe deer may not constitute a profitable prey type for wolves if they must actively be searched for at lower densities. In this scenario wolves should only prey on roe deer opportunistically when encountered [59]. An alternative hypothesis is that there are indirect effects of roe deer population density on their vulnerability through intra-specific competition and limitation of resources [60, 61].

A higher population density may also lead to behavioural effects on roe deer, by increasing their tendency to aggregate in numbers [61], potentially making their location more predictable to wolves. Previous studies have shown that large predators are able to adjust the utilisation of their territory in response to the location of the selected prey type [8, 62]. Therefore one could hypothesise that wolves at the intra-territory level should spend more time in areas with a higher density of roe deer, thereby reducing their search distance and increasing the profitability of this prey [63]. Theoretically, predation on moose would then be further limited as wolves may experience lowered encounter rates with moose in roe deer habitat [64, 65]. However, this hypothesis was not supported in an investigation of wolf predation and space use in Scandinavia [59]. That study used a subset of the data available for the current study in order to investigate the effect of prey density, wolf distribution, and landscape structure on the probability of occurrence of a wolf kill for moose and roe deer. The authors found that wolves were more likely to kill roe deer in areas of their territory where they spent more time searching for prey (i.e., handling time excluded) but that roe deer density per see was not an important factor affecting their space use at the intra-territorial level. In this sense, these two studies may seem to yield contradictory results. However, whereas Gervasi et al. [59] modelled the probability of a site being a wolf kill site (as compared to randomly distributed locations within the wolf territory), the current study tested the probability for a known wolf kill being either roe deer or moose and how this was related to the density of each prey species. These studies are therefore complementary and show that 1) wolves do not spend their time in proportion to the spatial variation in roe deer density [59] and, 2) when killing an ungulate prey, this is far more likely to be a roe deer than moose in areas of high roe deer density (this study).

Earlier research on prey selection of wolves has not yielded consistent results with variable selection patterns among systems including multiple prey species [8, 20, 22, 28, 66]. In Europe, Jędrzejewski et al. [20] and Novak et al. [67] found that red deer was the main prey of wolves in Poland, that this species was selected for, and there was a strong positive association between the proportion of red deer in wolf diet and their population density. In Italy, Mattiolo et al. [68] found that wild boar constituted the main prey for wolves, and that this was the preferred prey species, but that this preference was inversely related to their population density. Roe deer, red deer and fallow deer were generally avoided and there was no dietary response to changes in prey density of either primary or secondary prey species. Other studies in Europe [69, 70, 71] support the results from our study, i.e., that when roe deer are available at sufficiently high densities, they usually constitute a major part of the prey and sometimes become the preferred prey species of wolves. However, the prey community characteristics in Scandinavia are different from those of central and southern Europe where large ungulates like moose are not present. In Italy [71], the average roe deer density was >3 times higher than found in the wolf territory with the highest density in Scandinavia, whereas the study in Poland [67] had average roe deer densities similar to the ones with the highest density in Scandinavia.

### Risk and effort

One might speculate how roe deer, differing moose in size by a factor of 6–12 depending on age class characteristics [34], may become the main predictor of wolf prey selection and govern prey choice of wolves. If only profitability in terms of prey biomass yield per kill were important for prey selection, wolves should at similar densities select moose over roe deer. A previous study of wolf hunting success in Scandinavia showed that the ratio of successful/unsuccessful attacks was equal for moose and roe deer [72]. The current study showed that roe deer and moose were equally likely to be killed by wolves at similar density. Whereas killing moose is generally associated with a larger effort involved and an increased risk of injury to the predator [12, 26, 73] the smaller roe deer is unlikely to pose a risk to wolves. It is therefore likely that the risk of injury is an important factor for the decision of wolves to select roe deer when present at sufficiently high densities. A risk-aversive strategy among Scandinavian wolves is further supported by the strong preference for young-of-the-year when preying on moose [33, 34]. Results from this study also showed that pairs of wolves were more than two times as likely, as compared to packs, to select roe deer over moose at comparable densities of moose and roe deer. This may be an adaptive strategy because the proportion of prey biomass lost to scavengers will increase with the size of prey and decrease with increased group size of the predator [74].

### Effects of snow conditions and time of year

In the boreal regions of the northern hemisphere, seasonal variation in climate and forage conditions is known to have a major impact on body condition of ungulates [31, 56, 75]. Snow conditions may both restrict the access to food and hamper movements, ultimately leading to reduced body condition [76]. In particular, as the winter proceeds and snow accumulates we would expect the smaller roe deer to be more negatively affected than moose, because of their smaller body reserves and due to a greater hindrance of their mobility [8, 77]. However, our results showed that neither winter conditions in terms of snow depth nor the time during winter was important for the wolves’ selection of roe deer versus moose. These results were surprising because other studies have found a strong relationship between snow conditions and the selection of different prey species [8, 23, 78] and our study area seems to have included enough variation in snow depth assumed to be important for the vulnerability of small-sized deer to predation [66, 79, 80]. Previous studies in Scandinavia have shown that increased snow depth resulted in both a higher proportion of a vulnerable segment of the moose population (calves) in wolf kill [59] as well as reduced chase distances of wolves on both moose and roe deer [72]. Obviously, we have currently an insufficient understanding of how winter conditions may affect prey vulnerability and the selection pattern of wolves in Scandinavia.

### Impact of wolves on prey population dynamics

The pattern of prey selection of predators and its relationship to density (i.e., functional response) of each prey species is central for the capacity of the predator to control or impact on prey populations [2, 5]. In this two-prey system, the functional response of wolves was mainly dependent on the density of the smaller prey species, the roe deer. Although human harvest is the dominant mortality factor for these ungulate species in our system [81, 82], our results are likely to have important implications for the wolf-prey dynamics in areas being colonized by the expanding wolf population. This is because these areas include alternative prey species such as roe deer, red deer, fallow deer and wild boar at much higher densities than so far has been present in the wolf distribution area. One prediction is therefore that wolf predation may constitute an important factor for the dynamics of these ungulate populations. Because all ungulate species are important game species in Scandinavia, gaining quantitative knowledge on how wolves will impact on the game communities is also important for the management of sustainable harvest. Wolf predation can be a significant limiting factor of prey population growth rate [17, 38, 42], because winter predation from wolves on moose is only to a minor part compensatory to starvation mortality [31]. Furthermore, the abundance of preferred prey species may have a strong influence on predator density [28, 83]. In fact, wolf territory sizes are strongly negatively related to the density of roe deer, but show no relation to moose density, among wolf packs in Scandinavia [37]. This means that the density of roe deer is closely linked to both the functional and the numerical response of wolves, two factors that govern the total predation impact of wolves on their prey. In conclusion, the presence of alternative prey species such as roe deer should result in a relaxed predation rate on the moose population [38], which in turn would be important for the harvestable surplus of both roe deer and moose. This also highlights the need for management to adopt an ecosystem approach that goes beyond simple two-species models, to instead including multiple interacting species in both the prey- and predator community [84].
